# Genomic Selection Improves Response to Selection in Resilience by Exploiting Genotype by Environment Interactions

**DOI:** 10.3389/fgene.2016.00178

**Published:** 2016-10-13

**Authors:** Han A. Mulder

**Affiliations:** Animal Breeding and Genomics Centre, Wageningen University and Research CentreWageningen, Netherlands

**Keywords:** genotype by environment interaction, breeding programs, response to selection, accuracy, genomic selection, resilience, reaction norm model

## Abstract

Genotype by environment interactions (GxE) are very common in livestock and hamper genetic improvement. On the other hand, GxE is a source of genetic variation: genetic variation in response to environment, e.g., environmental perturbations such as heat stress or disease. In livestock breeding, there is tendency to ignore GxE because of increased complexity of models for genetic evaluations and lack of accuracy in extreme environments. GxE, however, creates opportunities to increase resilience of animals toward environmental perturbations. The main aim of the paper is to investigate to which extent GxE can be exploited with traditional and genomic selection methods. Furthermore, we investigated the benefit of reaction norm (RN) models compared to conventional methods ignoring GxE. The questions were addressed with selection index theory. GxE was modeled according to a linear RN model in which the environmental gradient is the contemporary group mean. Economic values were based on linear and non-linear profit equations. Accuracies of environment-specific (G)EBV were highest in intermediate environments and lowest in extreme environments. RN models had higher accuracies of (G)EBV in extreme environments than conventional models ignoring GxE. Genomic selection always resulted in higher response to selection in all environments than sib or progeny testing schemes. The increase in response was with genomic selection between 9 and 140% compared to sib testing and between 11 and 114% compared to progeny testing when the reference population consisted of 1 million animals across all environments. When the aim was to decrease environmental sensitivity, the response in slope of the RN model with genomic selection was between 1.09 and 319 times larger than with sib or progeny testing and in the right direction in contrast to sib and progeny testing that still increased environmental sensitivity. This shows that genomic selection with large reference populations offers great opportunities to exploit GxE to increase resilience of animals.

## Introduction

Genotype by environment interaction (GxE) has been an issue in animal breeding for a very long time. Falconer (1952) invented the concept of genetic correlation between performance in different environments as a measure of GxE. Consequences of GxE for breeding programs have been studied by several researchers in the past (James, 1961; Dickerson, 1962; Banos and Smith, 1991; Smith and Banos, 1991) and in more recent years (Mulder and Bijma, 2005, 2006; Mulder et al., 2006; Buch et al., 2009). GxE often reduces response to selection (Mulder and Bijma, 2005). If GxE is not strong, recording relatives in different environments can alleviate the reduction in response to selection caused by GxE. If GxE is strong, i.e., a genetic correlation between environments lower than 0.6–0.7, different breeding programs are needed (Mulder et al., 2006). In such cases environments are usually considered as being different groups of farms based on geographical location (Zwald et al., 2001; Fikse et al., 2003), milking system (Mulder et al., 2004), grazing/no grazing (Boettcher et al., 2003; Kearney et al., 2004a,b; van der Laak et al., 2016) or organic/conventional farms (Nauta et al., 2006). There is however also GxE within farms between periods with and without stress, e.g., heat stress (Ravagnolo and Misztal, 2000; Zumbach et al., 2008; Bloemhof et al., 2012) or due to disease outbreaks (Herrero-Medrano et al., 2015). This within-farm GxE requires a different attitude in breeding programs toward improving performance in stressed and unstressed periods, i.e., to increase resilience.

There is a long-standing desire to increase resilience or robustness of animals. In that sense, GxE is no longer a burden because of lower response to selection, but a blessing because it is a source of genetic variation for adaptation to environments. In cases like heat stress or disease outbreaks, reaction norm (RN) models are better able to deal with the continuity of the environment than multivariate models or character state models, because fewer parameters need to be estimated and interpolation is possible (Kirkpatrick and Bataillon, 1999; De Jong and Bijma, 2002). Genetic variation in slope of a linear RN model can be considered as genetic variation in environmental sensitivity (ES). Kolmodin et al. (2003) showed that mass selection would increase ES, while Kolmodin and Bijma (2004) showed that ES can be changed by mass selection depending on the position of the selection environment with respect to the environmental gradient. Sae-Lim et al. (2015b) showed that the heritability of ES in aquaculture species is generally low between 0 and 0.15. Sae-Lim et al. (2015b) derived the co-heritability of ES and found that the coheritability is generally between −0.1 and 0.1 depending on the RN-parameters. As described in Falconer and Mackay (1996), when computing the response to mass selection, substituting co-heritability for heritability gives the correlated response to mass selection. Theoretically, the coheritability of ES is between 0 and −0.1 when the genetic correlation between two environments is higher or equal to 0.67, the heritability of the trait is 0.3 and no heterogeneity of additive genetic variance. In most cases, genetic correlations between environments are very high, above 0.8, in most livestock species, but lower between extreme environments when using a RN model (Zumbach et al., 2008; Herrero-Medrano et al., 2015) and also lower in aquaculture species (Sae-Lim et al., 2015a). This would indicate that ES can be considered as a trait with a low heritability and therefore accuracy of selection is limited. Furthermore, the accuracy of selection for performance in extreme environments is expected to be low, because such environments tend to have limited information for breeding value estimation. Especially for such cases, genomic selection can enhance response to selection. Using cross-validation, Silva et al. (2014) showed that a genomic RN model increased accuracy of EBV compared to pedigree-based EBV, especially in extreme environments. Furthermore, they showed clear advantage of a RN model compared to a conventional model ignoring GxE. Also, Rashidi et al. (2014) showed an increase in accuracy of sow effects, i.e., combined effect of breeding value and permanent environmental effect because of lack of pedigree, using a RN model compared to a bivariate model (periods with or without disease outbreaks) or a conventional model ignoring GxE. In another study, the improvement in accuracy of EBV comparing a RN model with different environmental parameters to a conventional model ignoring GxE was 3–8% (Rashidi, 2016). From a theoretical point of view it is unknown what the advantage is of RN models compared to conventional models. Furthermore, it is unknown by how much genomic selection can increase response to selection across environments compared to traditional sib or progeny testing schemes in the presence of GxE.

The main aim of this study was therefore to investigate how to exploit GxE to increase resilience with traditional and genomic selection methods. The first question was to investigate the benefit of using RN models in breeding value estimation compared to conventional methods ignoring GxE. The second question was to investigate the benefit of genomic selection compared to traditional sib or progeny testing schemes to exploit GxE. The questions were addressed with selection index theory. Economic values were derived using derivatives of linear and non-linear profit equations.

## Materials and methods

### Quantitative genetic framework

Here we assumed that the underlying model was a linear RN model generating GxE between pairs of environments. The trait considered was a sex-linked quantitative trait like milk production in cattle or litter size in pigs. A simple quantitative genetic model with additive genetic effects and environmental effects was considered, assuming that the trait was only measured once per animal and in absence of non-additive genetic effects and common environmental effects:

(1) $$P=\mu +bx+A_{int}+A_{sl}x+E$$

where *P* is the phenotype, μ is the overall mean, *b* is the fixed slope of the RN, *x* is the environmental parameter related to environment, *A*_*int*_ is the breeding value for intercept of the RN, *A*_*sl*_ is the breeding value for slope of the RN, and *E* is the residual environmental effect. The environmental parameter *x* was assumed to be continuous and related to the degree of environmental disturbance, for instance due to disease or heat stress. Effectively, the environmental parameter *x* was a contemporary group mean such as herd-year-season as a deviation from a herd-year effect. The environmental parameter *x* was assumed normally distributed and standardized with mean zero and variance 1 (*x* ~ *N*(0, 1)). The intercept of the RN was therefore in the average environment. The breeding values *A*_*int*_ and *A*_*sl*_ were bivariate normally distributed as $\left[\begin{array}{c} a_{int}\\ a_{sl} \end{array}\right]\sim MVN\left(\left[\begin{array}{c} 0\\ 0 \end{array}\right],A⊗\left[\begin{array}{cc} \sigma_{A_{int}}^{2} & \sigma_{A_{int},A_{sl}}\\ \sigma_{A_{int},A_{sl}} & \sigma_{A_{sl}}^{2} \end{array}\right]\right)$, where **A** is the numerator relationship matrix, $\sigma_{A_{int}}^{2}$, $\sigma_{A_{sl}}^{2}$ and $\sigma_{A_{int},A_{sl}}$ are the additive genetic variances and covariance between the two breeding values. Because RN models can be interchanged with multivariate models (De Jong and Bijma, 2002; Sae-Lim et al., 2015b), we used here the multivariate approach to predict responses to selection in different environments. We divided the area of the normal distribution between −2 and 2 in 11 parts plus the parts lower than −2 and higher than 2; in total 13 environments. The parts within −2 and 2 had equal intervals of *x*. The proportion of animals per environment was determined as the area under the curve of a normal distribution. The continuity of the environment was mimicked with 13 environments. Preliminary results showed hardly any changes in response to selection per environment when increasing the number of environments. Table 1 shows the 13 defined environments and the distribution of the reference population for genomic selection and the progeny or sibs for progeny and sib testing across the 13 environments.

**Table 1:** **The average environment and distribution of animals in the reference population or progeny across 13 environments assuming a normally distributed environmental parameter *x* and basic number of animals in the reference population (5000) and number of progeny (100)**.

**Environment**	***x* lower bound**	***x* higher bound**	**Proportion of environment**	***x* average**	**Number of animals reference population**	**Number of progeny**
1	−∞	−2.00	0.02	−2.37	113.75	2.28
2	−2.00	−1.64	0.03	−1.80	140.66	2.81
3	−1.64	−1.27	0.05	−1.44	253.38	5.07
4	−1.27	−0.91	0.08	−1.08	400.47	8.01
5	−0.91	−0.55	0.11	−0.72	555.35	11.11
6	0.55	−0.18	0.14	−0.36	675.71	13.51
7	−0.18	0.18	0.14	0.00	721.37	14.43
8	0.18	0.55	0.14	0.36	675.71	13.51
9	0.55	0.91	0.11	0.72	555.35	11.11
10	0.91	1.27	0.08	1.08	400.47	8.01
11	1.27	1.64	0.05	1.44	253.38	5.07
12	1.64	2.00	0.03	1.80	140.66	2.81
13	2.00	∞	0.02	2.37	113.75	2.27

### Defining the breeding goal

Because we approximated the RN model with a multivariate selection index, we defined the breeding goal *H* as:

(2) $$H=v'a=v_{1}A_{1}+v_{2}A_{2}\ldots +v_{n}A_{n}$$

where *v*_*i*_ is the economic value of environment *i* and *A*_*i*_ is the breeding value in environment *i*. We used in this study two breeding goals: (1) a proportional breeding goal with the economic values equal to the frequencies of animals in each environment according to the normal distribution, i.e., a linear profit equation and (2) a resilience breeding goal using a non-linear profit equation illustrating the law of diminishing returns. It was called a resilience breeding goal, because more weight was put on performance in low environments than on performance in high environments, basically aiming to diminish ES. For breeding goal 2, we used the equation presented by Eskridge and Johnson (1991). The equation was used in Eskridge and Johnson (1991) as a utility function reflecting the degree of risk aversion of farmers with respect to yield in plant varieties. Here, we use this equation as an example of a non-linear profit equation reflecting the law of diminishing returns. The profit equation used her was:

(3) Profit=1−exp(−0.3∗P)

**Figure 1:**
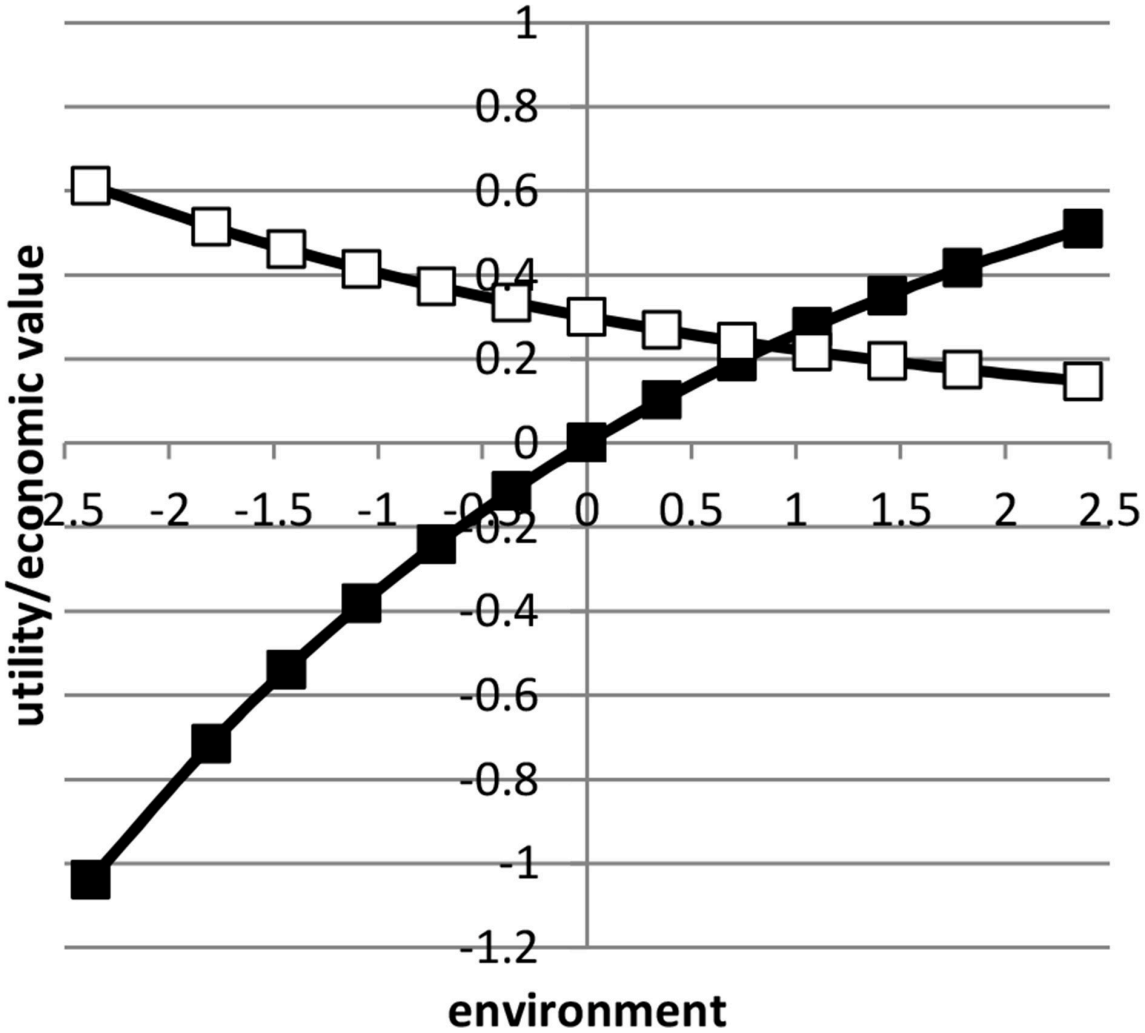
**The profit equation (black blocks) and the economic value (open blocks) as a function of environment**.

The economic value per environment can be derived for different levels of *P*, i.e., different levels of *x*:

(4) $$v_{i}=\frac{dProfit}{dx_{i}}=0.3\exp\text{​}(-0.3E\left(P_{i}\right))=0.3\exp\text{​}\left(-0.3(\mu +b\bar{x_{\iota }})\right)$$

where $\bar{x_{\iota }}$ was the average value of *x* in environment *i* based on a normal distribution. Here, we assumed μ = 0 and *b* = 1. Figure 1 shows the profit equation and its derivative as a function of the environmental gradient *x*. By using the value 0.3 in Equation (3), the economic values in the lowest and highest environment approximately differed by a factor four.

### Selection index framework

#### Accuracy of EBV per environment

The first objective was to quantify the accuracy of progeny-based and genomic-based environment-specific breeding values. Progeny and the reference population were assumed to be distributed across the environments according to frequencies based on the normal distribution (Table 1). To calculate the accuracy of EBV per environment, we used selection index theory and set the breeding goal to the environment of interest (*v*_*i*_ = 1, economic value in environment *i*) and setting all other economic values to zero. For progeny information, the index consisted of progeny averages in each environment:

(5) Iprog = b1P1¯+…+bnPn¯ = bprog'xprog

where *b*_*i*_ are selection index weights for the progeny mean in environment *i*, $\bar{P_{\iota }}$ is the average phenotype of half-sib progeny in environment *i*. The optimal selection index weights **b**_***prog***_ were calculated using selection index theory (Hazel, 1943):

(6) $$b_{prog}=P_{prog}^{-1}G_{prog}v$$

where **P**_***prog***_ is the variance-covariance matrix of the information sources in the selection index, **G**_***prog***_ is the covariance matrix between information sources in the selection index and the breeding values in the breeding goal and **v** is the vector with economic values for each environment. The matrix **P**_***prog***_ was calculated as:

(7) $$P_{prog}\;=\;\left[\begin{array}{ccc} \text{var}(\bar{P_{1}}) & \cdots & \text{cov}(\bar{P_{1}},\bar{P_{n}})\\ \vdots & \ddots & \vdots \\ \text{cov}(\bar{P_{1}},\bar{P_{n}}) & \cdots & \text{var}(\bar{P_{n}}) \end{array}\right]$$

where $\text{var}\left(\bar{P_{\iota }}\right)$ and $\text{cov}\left(\bar{P_{\iota }},\bar{P_{j}}\right)$ were calculated as:

(8) $$\text{var}(\bar{P_{\iota }})=\left(\text{​}\begin{array}{c} \sigma_{A_{int}}^{2}+2x_{i}\sigma_{A_{int},A_{sl}}+x_{i}^{2}\sigma_{A_{sl}}^{2}+\sigma_{e}^{2}+\\ \left(\left(n_{i}-1\right)*0.25*(\sigma_{A_{int}}^{2}+2x_{i}\sigma_{A_{int},A_{sl}}+x_{i}^{2}\sigma_{A_{sl}}^{2})\right) \end{array}/n_{i}\right)$$

and

(9) $$\text{cov}\left(\bar{P_{\iota }},\bar{P_{j}}\right)\;=\;0.25*(\sigma_{A_{int}}^{2}+(x_{i}+x_{j})\sigma_{A_{int},A_{sl}}+x_{i}x_{j}\sigma_{A_{sl}}^{2})$$

The matrix **G**_***prog***_ was calculated as:

(10) $$\text{cov}\left(\bar{P_{i}},A_{j}\right)=0.5*(\sigma_{A_{int}}^{2}+(x_{i}+x_{j})\sigma_{A_{int},A_{sl}}+x_{i}x_{j}\sigma_{A_{sl}}^{2})$$

For genomic selection, we assumed that in each environment a genomic EBV (GEBV) was estimated with a univariate model using only the reference population of that environment. For convenience, these GEBV were scaled toward a variance of one. The accuracy of each GEBV was calculated following Daetwyler et al. (2008):

(11) $$r_{i}\;=\;\sqrt{\frac{N_{i}h_{i}^{2}}{N_{i}h_{i}^{2}+M_{e}}}$$

where $h_{i}^{2}$ is the heritability in environment *i* and calculated as $h_{i}^{2}\;=\;(\sigma_{A_{int}}^{2}+2x_{i}\sigma_{A_{int},A_{sl}}+x_{i}^{2}\sigma_{A_{sl}}^{2})/(\sigma_{A_{int}}^{2}+2x_{i}\sigma_{A_{int},A_{sl}}+x_{i}^{2}\sigma_{A_{sl}}^{2}+\sigma_{e}^{2}),N_{i}$ is the size of the reference population in environment *i* and *M*_*e*_ is the effective number of chromosome segments, which was assumed constant across environments. Subsequently, the GEBV of all environments were combined into an index:

(12) IGS=b1GEBV1+…+bnGEBVn=bGS'xGS

where **b**_***GS***_ was calculated using Equation (6) and replacing **P**_***prog***_ and **G**_***prog***_ by **P**_***GS***_ and **G**_***GS***_. The matrix **P**_***GS***_ was calculated as:

(13) $$P_{GS}=\left[\begin{array}{ccc} 1.0 & \cdots & r_{g,ij}r_{i}r_{j}\\ \vdots & \ddots & \vdots \\ r_{g,ij}r_{i}r_{j} & \cdots & 1.0 \end{array}\right]$$

where *r*_*g, ij*_ is the genetic correlation between environment *i* and *j* and *r*_*i*_ and *r*_*j*_ are the accuracies of GEBV in environment *i* and *j*. The matrix **G**_***GS***_ was calculated as:

(14) $$\text{cov}\left(GEBV_{i},A_{j}\right)\;=\;r_{g,ij}r_{i}\sqrt{\sigma_{A_{int}}^{2}+2x_{j}\sigma_{A_{int},A_{sl}}+x_{j}^{2}\sigma_{A_{sl}}^{2}}$$

The method is in essence identical to Wientjes et al. (2016) for one breed, but differs slightly in mathematical expressions. The accuracy of EBV or GEBV per environment combining the information across environments was finally calculated as:

(15) $$r_{i,index}\;=\;\frac{b^{\prime }g_{i}}{\sigma_{I}\sqrt{\sigma_{A_{int}}^{2}+2x_{i}\sigma_{A_{int},A_{sl}}+x_{i}^{2}\sigma_{A_{sl}}^{2}}}$$

where $\sigma_{I}=\sqrt{{\text{b}}^{\prime }\text{P}\text{b}}$, i.e., the standard deviation of the index.

When a conventional model was used for breeding value estimation ignoring GxE, all elements of **P**_***prog***_ were averaged using the weights $\left(n_{i}n_{j}\right)/{\left(\sum_{i=1}^{i=n}n_{i}\right)}^{2}$, where *n*_*i*_ is the number of progeny in environment *i*, and all elements of **G**_***prog***_ were averaged per column to be able to calculate accuracies per environment. For genomic selection, we combined the univariate GEBV of each environment into an overall index to maximize response in *A*_*int*_ ignoring GxE using $P_{GS,\;conv}\;=\;\left[\begin{array}{ccc} 1.0 & \cdots & r_{i}r_{j}\\ \vdots & \ddots & \vdots \\ r_{i}r_{j} & \cdots & 1.0 \end{array}\right]$ and *g*_*i*_ = *r*_*i*_. Subsequently, Equation (15) was used to calculate *r*_*i, index*_, setting the genetic variance in environment *i* to one $\left(\sigma_{A_{int}}^{2}+2x_{i}\sigma_{A_{int},A_{sl}}+x_{i}^{2}\sigma_{A_{sl}}^{2}=1.0\right)$.

### Pseudo-BLUP selection index

**Figure 2:**
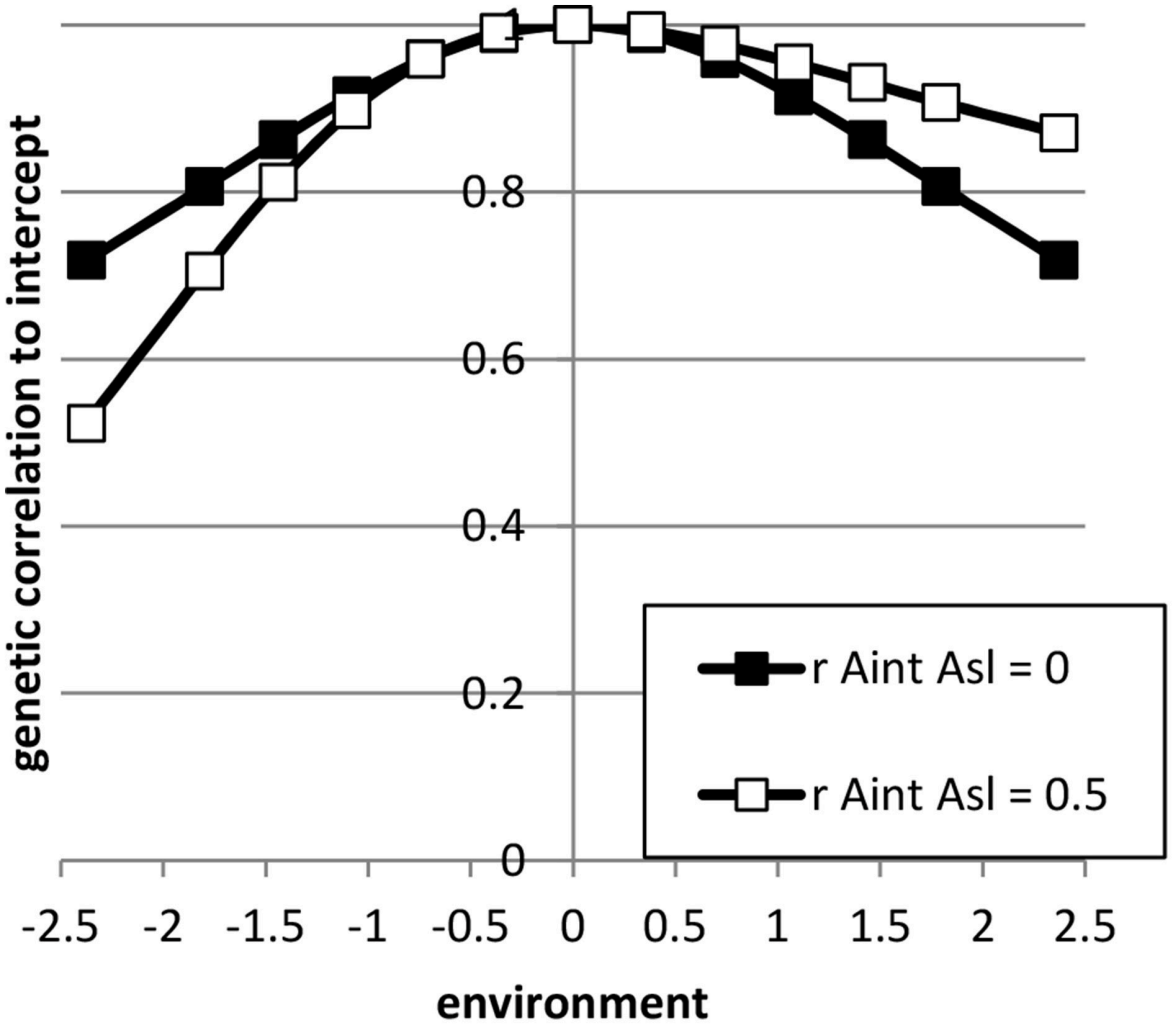
**The genetic correlation between performance in a certain environment and the average environment (= intercept) for the sets of reaction norm parameter values used**. (see Table 2).

The expressions 5 till 15 were extended to a pseudo-BLUP index. The pseudo-BLUP selection index approximates BLUP selection by including pedigree information in the selection index (Wray and Hill, 1989; Dekkers, 1992; Villanueva et al., 1993). Furthermore, the pseudo-BLUP selection index accounts for reduction of genetic variance due to selection (Bulmer, 1971). For sib or progeny testing equations were used as presented in Mulder and Bijma (2005). For sib testing of sires and dams, we used information of half-sibs in each environment and in addition full-sib information in the nucleus environment, which was considered the best environment (*x* = 2.37). Furthermore, female selection candidates had own performance in the best environment. Because BLUP selection was assumed, we included the EBV of sires and dams in the best and worst environment, as well as the EBV of the dams of the half-sibs. We could not use EBV for all environments, because of singularities in matrices due to very high correlations between EBV of different environments. For progeny testing, we used for females the same information as for sib testing; for males we used the same information as for sib testing, except half-sibs were replaced by half-sib progeny in each environment. For genomic selection, the pseudo-BLUP index used GEBV as information using the Equations (13) and (14). Response to selection per environment was calculated following Mulder and Bijma (2005) using relative generation intervals of 1 for sib testing and genomic selection and 1.6 for sires in an efficient progeny testing scheme. Response to selection in intercept of the RN was the response in the average environment, while the response in the slope of the RN was calculated as *R*_*A*_*sl*__ = (*R*_*i*_ − *R*_*average*_)/*x*_*i*_, where *R*_*i*_ is the response in environment *i* (Note that, *i* should be different than the average environment, but it does not matter which other environment is used because the RN is linear). Selection intensities were calculated assuming a finite population of selection candidates and corrected for correlated index values among relatives (Hill, 1976; Meuwissen, 1991). The selected proportions in males and females were assumed 5% and 20%, respectively. The input parameters are summarized in Table 2; Figure 2 shows the genetic correlation between a certain environment and the average environment for the two sets of genetic parameters used.

**Table 2:** **Values of parameters used in calculating response to selection in sib testing, progeny testing schemes, and genomic selection schemes: basic parameters and range of values used in alternative breeding schemes**.

**Parameter**	**Basic**	**Alternatives range**
Additive genetic variance intercept: $\sigma_{A_{int}}^{2}$	0.3	0.1, 0.5
Additive genetic variance slope: $\sigma_{A_{sl}}^{2}$	0.05	
Genetic correlation intercept slope *r_A_int__*, *A*_*sl*_	0	0.5
Residual variance: $\sigma_{e}^{2}$	0.7	
Proportion of selected sires	0.05	
Proportion of selected dams	0.20	
Number of progeny per dam	10	
Number of animals in nucleus	2000	
Number of commercial half-sibs/half-sib progeny	100	
Reference population genomic selection (*N*)	5000	1,000,000
Relative generation interval progeny testing compared to genomic selection or sib testing	1.6	
Number of effective chromosome segments (*M_e_*)	1200	

## Results

### The benefit of a reaction norm model for estimating breeding values

**Figure 3:**
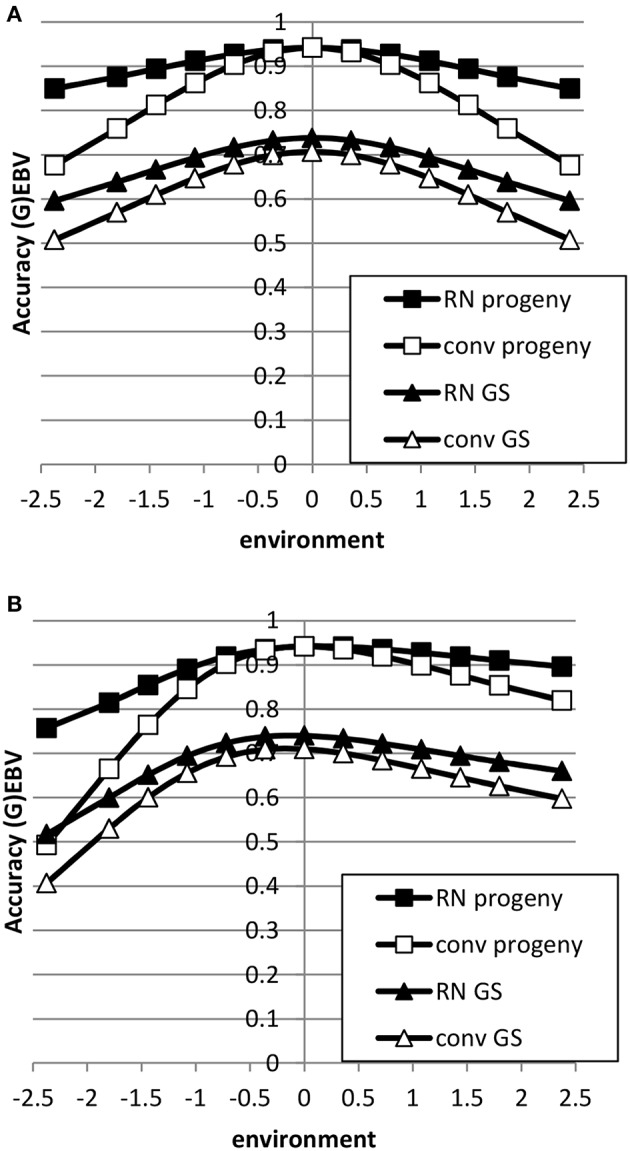
**Accuracy of progeny or genomic based breeding values as a function of environment with normally distributed progeny or reference populations across environments using a reaction norm model (RN) or a conventional model ignoring GxE (conv) (A: $r_{A_{int},A_{sl}}=0$; B: $r_{A_{int},A_{sl}}=0.5$)**. See Table 2 for parameter values.

**Figure 4:**
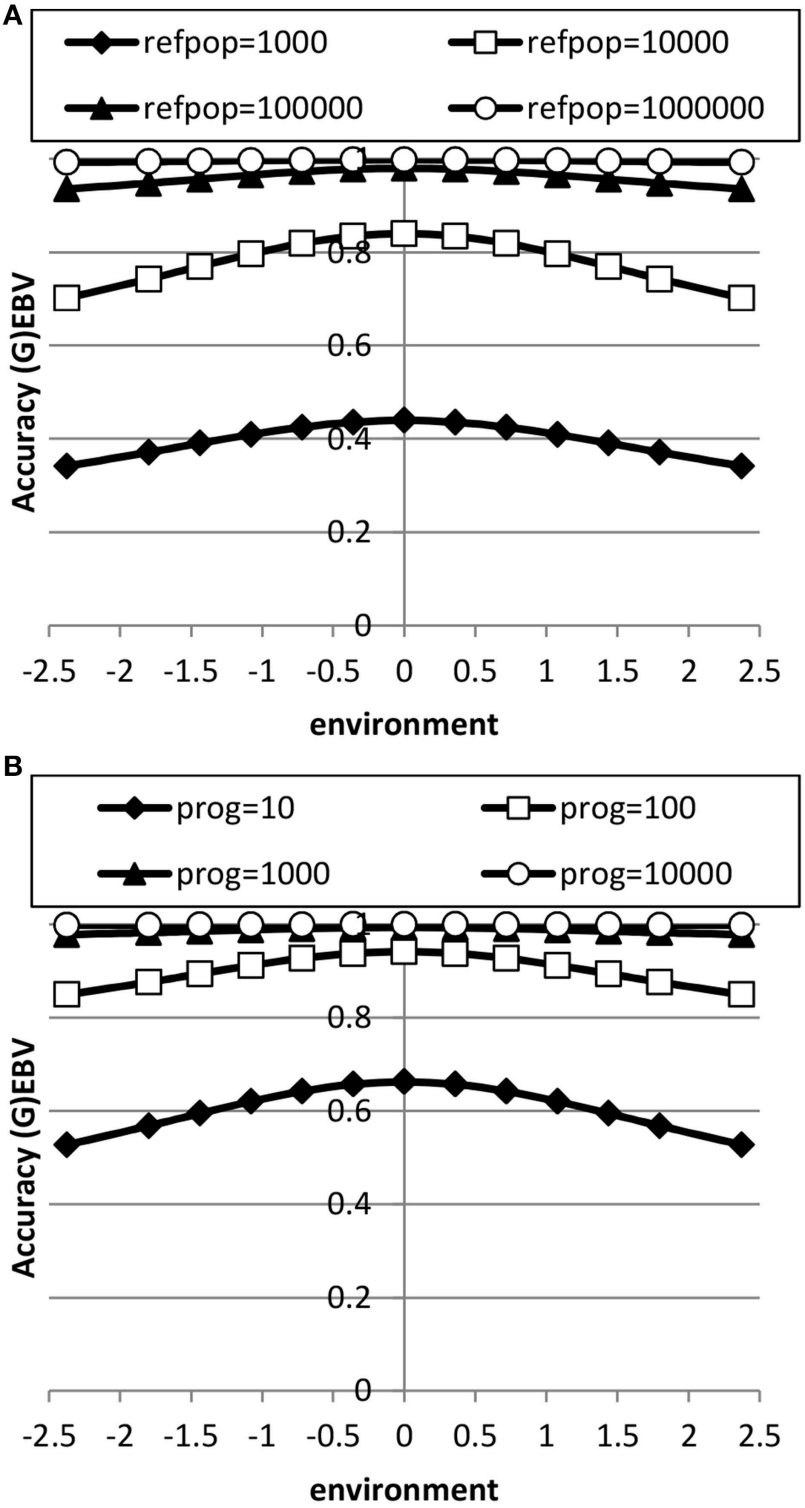
**Accuracy of genomic based (A) or progeny based (B) breeding values as a function of environment with normally distributed progeny (prog) or reference populations (refpop) across environments for different sizes of the reference population and number of progeny per sire using reaction norm models**. See Table 2 for paramater values.

The accuracy of EBV in extreme environments was higher with a RN model compared to a conventional model ignoring GxE for both genomic and progeny-based EBV (Figure 3). In the average environment, i.e., the intercept, the accuracy for both RN and conventional model were similar. When the genetic correlation between intercept and slope was 0.5, the difference in accuracy between the two models was largest in extremely negative/unfavorable environments. The EBVs based on progeny had higher accuracy than based on genomic prediction, but the accuracy of genomic prediction could be further improved with larger reference populations. When using a RN model, the increase in accuracy across environments with either increasing reference population for genomic EBV or with increasing number of progeny for progeny based EBV was similar as shown in Figure 4: in both cases accuracy reached unity in all environments with very large reference populations or numbers of progeny per sire. In summary, RN models resulted in higher accuracy of EBV in extreme environments than conventional models ignoring GxE.

### The benefit of genomic selection compared to sib or progeny information

**Figure 5:**
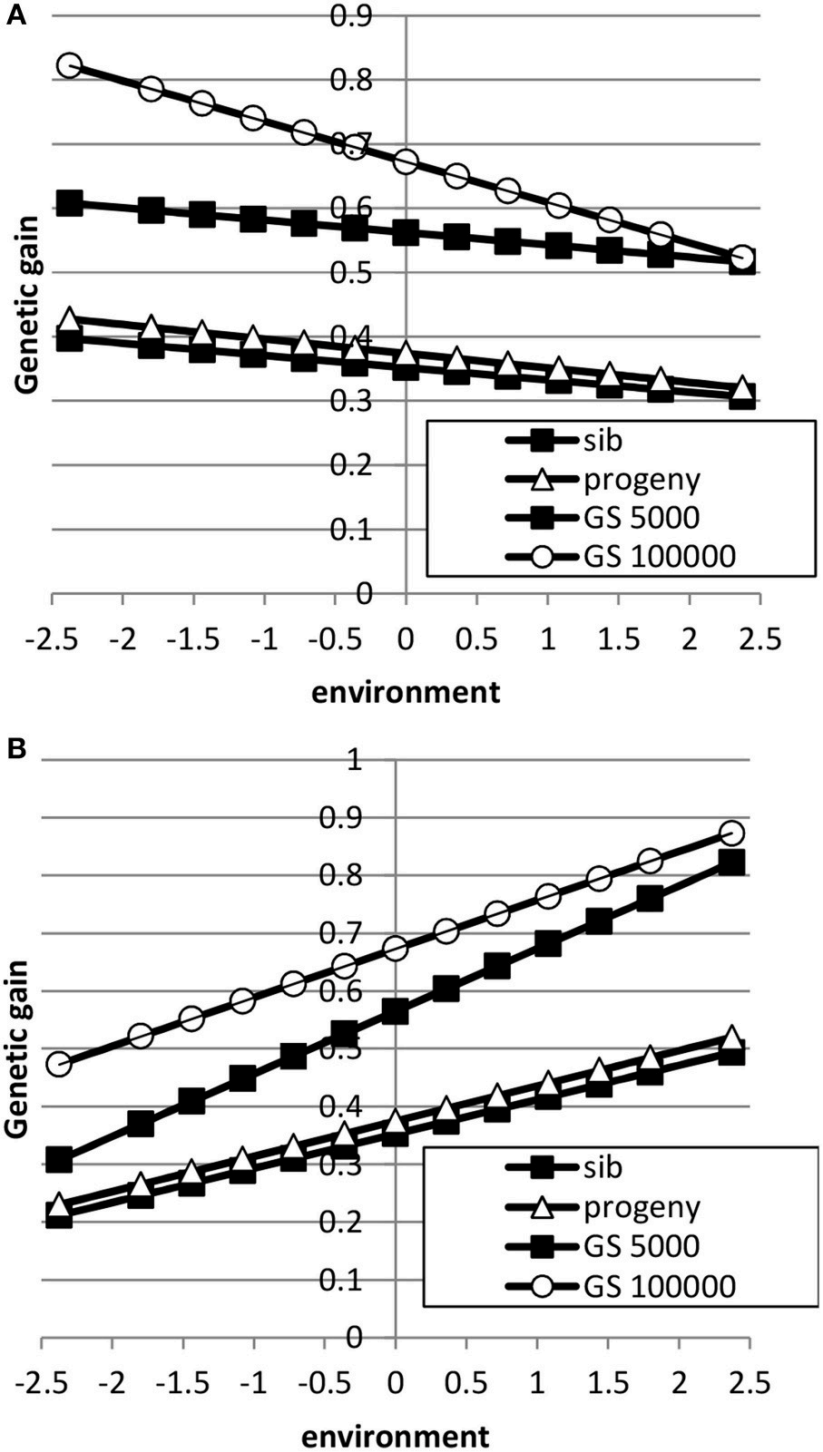
**Response to selection as a function of environment when the breeding goal is based on the profit equation (Equation 3) using sib testing (only half-sibs), progeny testing (progeny for sires; half-sibs for dams) or genomic selection when the reference population is 5000 or 1,000,000 animals (A: $r_{A_{int},A_{sl}}=0$; B: $r_{A_{int},A_{sl}}=0.5$)**. See Table 2 for parameter values.

**Figure 6:**
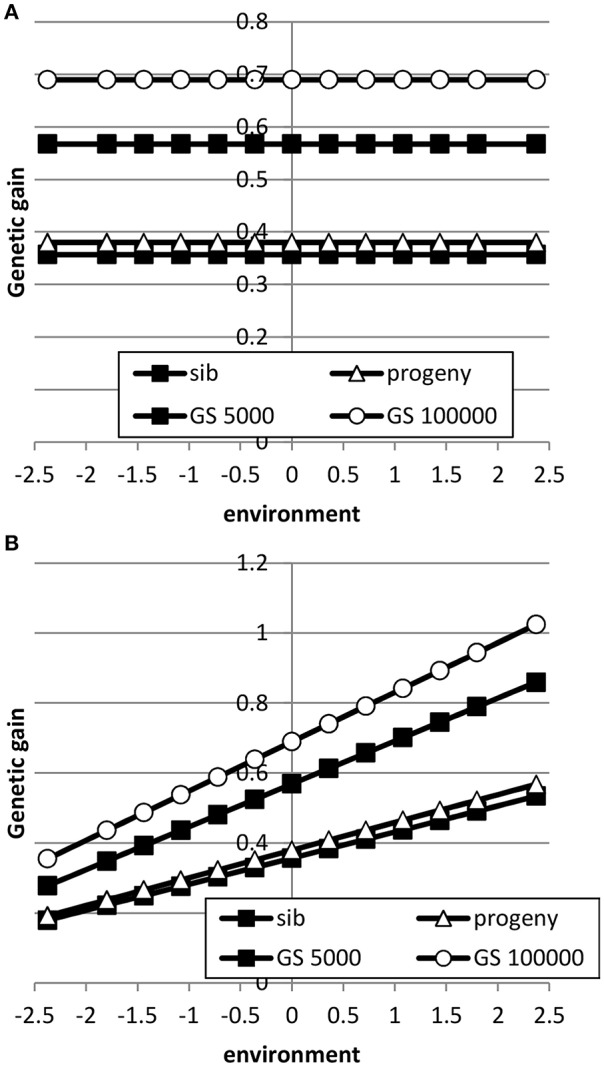
**Response to selection as a function of environment when each environment has a proportional economic value in the breeding goal according to the normal distribution using sib testing (only half-sibs), progeny testing (progeny for sires; half-sibs for dams) or genomic selection when the reference population is 5000 or 1,000,000 animals (A: $r_{A_{int},A_{sl}}=0$; B: $r_{A_{int},A_{sl}}=0.5$)**. See Table 2 for parameter values.

The breeding goal and the genetic correlation between intercept and slope had a large effect on response to selection as shown in Figures 5, 6. When the breeding goal had more emphasis on low environments, i.e., the resilience breeding goal, most response to selection was obtained in low environments when the genetic correlation between intercept and slope was zero. Genomic selection had higher response to selection than selection on sib or progeny information. Especially a very large reference population increased response to selection in low environments more than in high environments: i.e., the difference in response to selection became larger. When the genetic correlation between intercept and slope was 0.5, response to selection was still higher in high environments because of higher genetic variance in high environments than in low environments and the positive genetic correlation, which made it more difficult to have high response to selection in low environments. In that case, a very large reference population made response to selection more balanced across environments compared to a small reference population. A reference population of 5000 animals had a larger response to selection in high environments than in low environments, i.e., a steeper slope, than sib or progeny testing indicating that small reference populations would still increase ES. Results showed that genomic selection with a large reference population has better opportunity to increase performance in low environments than traditional breeding schemes.

When the breeding goal was proportional to the environment (Figure 6), response to selection was constant across environments for all types of selection when the genetic correlation between intercept and slope was zero. When the genetic correlation between intercept and slope was 0.5, response to selection was higher in high environments than in low environments, due to higher genetic variance in high environments than in low environments. Differences in response to selection between breeding schemes were smallest in low environments and highest in high environments. Increasing the reference population from 5000 to 1 million animals further increased ES because more response could be achieved in high environments with a higher genetic variance. It can be concluded that the response across environments is highly affected by the genetic correlation between intercept and slope.

### The benefit of genomic selection compared to traditional BLUP breeding schemes

Table 3 shows the benefit of genomic selection vs. traditional BLUP breeding schemes either based on sib or progeny testing using a pseudo-BLUP selection index. In all cases, genomic selection increased response to selection with increments between 9 and 140% compared to sib testing and between 10 and 114% compared to progeny testing schemes. The increase was largest in low environments and smallest in the high environments, because the breeding goal had more emphasis on low environments, whereas in sib testing and progeny testing schemes own performance on females and full-sib information was available in high environments, i.e., the nucleus environment. The advantage of genomic selection was larger for a heritability of 0.1 than for 0.3 and 0.5, especially with a large reference population. Traditional sib and progeny testing schemes obtained larger gain in high environments and increased ES, whereas genomic selection decreased ES. The response in slope, i.e., ES, of the RN model was with genomic selection between 1.09 and 319 times larger than with sib or progeny testing and in the right direction (Table 4). The response in intercept was equal to the response in the intermediate environment in Table 3. It should be noted that the values for slope of genomic selection compared to progeny testing were extremely large, because the response in slope was very close to zero for progeny testing and dividing by almost zero resulted in very large numbers. Results showed very good opportunities for genomic selection to increase resilience by exploiting genotype by environment interaction, especially with large reference populations.

**Table 3:** **Response to selection with genomic selection (GS) relative to response to selection of sib or progeny testing schemes based on a pseudo-BLUP selection index for 3 heritabilities^a^ and 2 sizes of the reference population for genomic selection^b^**.

	***h*^2^**	**Size of reference population**					
		**5000**			**1,000,000**		
		**Low**	**Middle**	**High**	**Low**	**Middle**	**High**
GS/sib	0.1	1.38	1.22	1.09	2.40	1.84	1.36
	0.3	1.60	1.37	1.17	2.17	1.64	1.19
	0.5	1.69	1.40	1.16	2.11	1.57	1.11
GS/progeny	0.1	1.23	1.17	1.10	2.14	1.76	1.37
	0.3	1.47	1.32	1.18	1.99	1.58	1.19
	0.5	1.55	1.34	1.16	1.94	1.51	1.11

a *heritability in average environment; due to change in genetic variance across environment and constant residual variance, heritabilities change over the range of environments*.

b *See Table 2 for used input*.

**Table 4:** **Response to selection in reaction norm parameters with genomic selection (GS) relative to response to selection of sib or progeny testing schemes based on a pseudo-BLUP selection index for 3 heritabilities^a^ and 2 sizes of the reference population for genomic selection^b^**.

	***h*^2^**	**Size of reference population**			
		**5000**		**1,000,000**	
		**BV intercept**	**BV slope**	**BV intercept**	**BV slope**
GS/sib	0.1	1.22	−1.09	1.84	−6.35
	0.3	1.37	−1.48	1.64	−4.86
	0.5	1.40	−1.47	1.57	−3.80
GS/progeny	0.1	1.17	−54.84	1.76	−319.34
	0.3	1.32	−3.60	1.58	−11.80
	0.5	1.34	−2.59	1.51	−6.68

a *Heritability in average environment; due to change in genetic variance across environment and constant residual variance, heritabilities change over the range of environments*.

b *See Table 2 for used input*.

## Discussion

### Methods and results

The aims of this study were to show the benefit of RN models compared to conventional models and to show the benefit of genomic selection to exploit GxE compared to traditional sib or progeny testing schemes. RN models gave higher accuracy of EBV in extreme environments, whereas accuracy was similar in average environments. Furthermore, genomic selection outperformed traditional breeding schemes by 9–140% across environments. Genomic selection was much better able to change ES, i.e., the slope of the RN model. With rapidly increasing reference populations in all livestock species, it becomes increasingly feasible to have high accuracy across all environments and makes environment-specific selection of sires for commercial use attractive, e.g., in cattle or in pigs.

Here, we found that the difference in accuracy of EBV between RN and a conventional model ignoring GxE increased when more progeny per sire were available or when the reference population was larger. The reason why RN models gave higher accuracy than conventional models is that conventional models ignore the genetic component due to slope and effectively only the intercept is used. With conventional models, the accuracy of the EBV in environment *i* becomes the accuracy of the EBV for intercept times the genetic correlation between environment *i* and the intercept. With RN models, the accuracy of the EBV in environment *i* is higher than with conventional models due to better exploiting the data in that environment and adjacent environments.

Here, we found that genomic selection with a reference population of 5000 animals outperformed traditional sib or progeny testing schemes (Figures 5, 6), while the accuracy of GEBV was lower than for progeny-based EBV (Figures 3, 4). The main reason is that the accuracy of selection either with sib testing both sexes or for sib-tested females in a progeny testing scheme is much lower than the accuracy of genomic selection. For instance, the accuracy of genomic selection (*r*_*IH*_, i.e., the accuracy of selection for the breeding goal) was 0.73 for both sexes with the proportional breeding goal when using the pseudo-BLUP selection index, whereas the accuracies of selection were 0.90 and 0.47 for males and females in a progeny testing scheme and 0.45 and 0.57 for males and females in a sib testing scheme. Furthermore, in a progeny testing scheme, the higher accuracy of progeny-tested males was offset by their longer generation interval. Thus, the higher average accuracy in both sexes and the short generation interval in both sexes lead to higher response to selection with genomic selection schemes compared to sib or progeny testing schemes.

The form of GxE was according to a linear RN model. In practice, the form of RN and the environmental parameter causing GxE are unknown. This requires statistical analysis and model comparison techniques such as Akaike's information criterion (AIC), Bayesian information criterion (BIC) or deviance information criterion (DIC) for Bayesian approaches. Furthermore, also cross-validation techniques can help. Selection index models such as developed in this study can help to set expectations for accuracies based on cross-validation, e.g., depending on the size of the reference population or the distribution of the reference population across environments.

Here, we extended the pseudo-BLUP selection index developed in Mulder and Bijma (2005) to multiple environments and to genomic selection equivalent of Wientjes et al. (2016). Sib testing and progeny testing when only sibs or progeny were used in breeding value estimation yielded very similar responses to selection across environments (Figures 5, 6). When nucleus information was included (Table 3), sib testing schemes increased response to selection in high environments more than in low environments, while progeny testing schemes had more balanced response to selection. Similarly, Mulder and Bijma (2005) found that sib testing was more sensitive to GxE between selection environment and production environment than progeny testing. Genomic selection was much better able to increase response to selection in low environments than traditional breeding schemes. It should be noted, however, that in practice genomic selection uses also pedigree information, whereas in our study we assumed that GEBV were only based on markers. Furthermore, we assumed that markers captured all genetic variance, while in practice high-density SNP chips and even sequence data may only capture part of the whole genetic variance. For instance, sequence data may suffer from cumulating sequencing and imputation errors, while high density SNP chips may capture only part of the genetic variation, because of incomplete linkage disequilibrium between QTL and SNPs. As a consequence, an accuracy of one may not be reached and our study may have over predicted the value of genomic selection compared to traditional selection methods. However, even when the maximum accuracy of genomic selection would be 0.9 or 0.95, genomic selection has still very good opportunities to exploit GxE and to decrease rather than increase ES.

We show that genomic selection is much better able to reduce ES than traditional selection schemes, which increased ES in agreement with previous studies (Kolmodin et al., 2003; van der Waaij, 2004). There are two reasons why genomic selection has better opportunities to exploit GxE than traditional selection methods: (1) less emphasis or no emphasis on own performance in optimal nucleus environments and (2) high accuracy of selection in unfavorable environments for both sexes. Especially with sib testing, e.g., in pigs and poultry, there is high emphasis on own performance in optimal nucleus environments. Genomic selection has the potential to move the emphasis to performance in commercial environments. Furthermore, with very large reference populations genomic selection has the potential to achieve accuracies of ~1 across the whole environmental range, whereas with traditional selection this is not feasible due to low amounts of information of relatives in extreme environments.

### Resilience and the breeding goal: diseases and climate change

Resilience is a trait which is loosely defined in many cases. Walker et al. (2004) defined resilience as “the capacity of a system to absorb disturbance and reorganize while undergoing change so as to still retain essentially the same function, structure, identity, and feedbacks,” while stability is the time required for an ecosystem to return to an equilibrium or steady-state following a perturbation (Holling, 1973). In animal breeding terms, we define resilience here as the ability of an animal to maintain performance despite perturbations and can therefore be translated into an animal with a low ES. Stability may be translated as decreasing variability or increasing uniformity. Selection for increased stability or uniformity will not be discussed here further as it is discussed elsewhere (Mulder et al., 2008; Hill and Mulder, 2010).

In this study, we defined a resilience breeding goal as one with more weight in the breeding goal on stressed environments, i.e., a low environment, and less weight on unstressed environments, i.e., high or optimal environments, such as the nucleus. Here, we defined the breeding goal consisting of performance in different environments; equivalently the breeding goal can be defined in terms of intercept and slope breeding values of RN. Clearly, improving resilience is only of importance if low environments have higher weight than high environments. Improving resilience has no economic value when environments have an effect on the overall breeding goal according to their frequencies and the frequency of environments follow a symmetric distribution, such as a normal distribution. Examples of breeding goals in which increasing resilience may be of importance are improving disease resilience and resilience to climatic environmental perturbations such as heat stress in livestock or variation in water temperature in aquaculture (Sae-Lim et al., 2016).

Improving disease resilience has been a research topic for decades. Two clear examples are resilience to nematode infections in sheep (Albers et al., 1987; Bisset and Morris, 1996) and resilience to PRRS in pigs (Rashidi et al., 2014; Herrero-Medrano et al., 2015). In the case of diseases, resilience may consist of two mechanisms: resistance and tolerance (Lewis et al., 2007; Kause, 2011; Doeschl-Wilson and Kyriazakis, 2012). Resistance is the ability of animals to restrict the invading pathogen's life cycle. A resistant animal will have minimum pathogen burden during an infection period. Tolerance is the animal's ability to minimize the symptoms of infection at a given pathogen burden. Rashidi (2016) showed that selection on the EBV for slope of a RN model, i.e., EBV for resilience, simultaneously improved resistance and tolerance, without the need to have records on pathogen burden. A clear example for improving resilience is to decrease the number of stillborn and mummified piglets during PRRS outbreaks both from economic and societal perspective. Herrero-Medrano et al. (2015) showed good heritabilities for number of lost piglets (the sum of stillborn and mummified piglets) during disease outbreaks.

Improving resilience to climatic environmental perturbations such as heat stress in livestock or variation in water temperature in aquaculture are other examples that have research interest. In both examples animals experience in part of the year stresses such as too high temperature or too low temperature. If climate change causes more fluctuations in temperature, breeding should aim for animals capable of handling more extreme temperatures, i.e., more resilient. The effect of climate change may be limited for livestock, but larger for aquaculture as fish are cold-blooded. In such cases genetic variation in response to temperature may help to breed fish better adapted to environments with larger fluctuations in water temperature (Sae-Lim et al., 2016).

### Optimizing genomic selection programs

Genomic selection is much better able to increase resilience than traditional breeding schemes provided that the reference population is well-spread across environments. Preliminary results showed hardly any difference in response when the reference population was either equally distributed across environments or according to proportions based on a normal distribution. However, if the whole reference population would be formed by elite farms with no environmental perturbations such as disease outbreaks, genomic selection would not be able to increase resilience. Therefore, it is crucial that the reference population should reflect as best as possible the environmental range that commercial progeny are expected to experience. For cattle, this would mean that ideally all farms in milk recording would genotype their cows. For crossbreeding schemes such as in pigs and poultry, it would be important to genotype many more commercial crossbred animals than currently is the case. Further, complications are that crossbreds provide information of only one parental haplotype per breed in case of two-way crosses and performance in different crossbred products may not be genetically the same trait. Clearly, here is room for optimization of cost-effective genotyping and phenotyping efforts.

## Conclusions

This study showed that RN models increased the accuracy of environment-specific EBV or GEBV compared to conventional models ignoring GxE. Large reference populations of 1 million animals increased accuracies up to one across the whole environmental range. Progeny testing schemes and sib testing performed very similar when the breeding goal was to increase performance across the whole environmental range. Genomic selection outperformed traditional breeding schemes, even with a reference population of 5000 animals. Genomic selection increased response to selection by 9% till 140% compared to sib testing and 11% till 114% compared to progeny testing. When the breeding goal was to increase performance in low environments more than in high environments, i.e., reduce ES, progeny testing increased ES less than sib testing. However, only genomic selection was able to reduce ES in absence of a genetic correlation between intercept and slope, especially with a reference population of 1 million animals. Therefore, it was concluded that genomic selection has much better ability to increase resilience and reduce ES.

## Author contributions

HM developed the selection index framework, performed the calculations, and wrote the manuscript.

### Conflict of interest statement

The author declares that the research was conducted in the absence of any commercial or financial relationships that could be construed as a potential conflict of interest.
